# Recruiting Diverse Dementia Family Caregivers: What Works for Which Groups?

**DOI:** 10.1093/geroni/igab046.1825

**Published:** 2021-12-17

**Authors:** Kyra Mendez, Valerie Cotter, Hae-Ra Han

**Affiliations:** 1 Johns Hopkins Bloomberg School of Public Health, Baltimore, Maryland, United States; 2 Johns Hopkins School of Nursing and School of Medicine, Baltimore, Maryland, United States; 3 Johns Hopkins School of Nursing, Baltimore, Maryland, United States

## Abstract

The purpose of this presentation is to compare success of recruitment methods by race/ethnicity, age, and kinship of dementia family caregivers. We conducted a cross-sectional study and recruited a convenience sample of dementia family caregivers using community-based and online methods. Recruitment success was tracked through survey questions, direct referrals, and community event sign-ups. Using chi-squared statistics, we examined the success of each method by caregiver race/ethnicity, age, and relationship to person with dementia (kinship). There were significant differences in recruitment source based on race/ethnicity, age, and kinship (P<.001). Specifically, referrals and newspaper advertisements were most successful for recruiting older (54 years+), White, non-Hispanic, and spousal or child caregivers; community events and reputable websites for recruiting older, minority, child caregivers; ResearchMatch for recruiting younger, minority, child/grandchild caregivers; and social media for recruiting younger, White, non-Hispanic, and child caregivers. Findings support the importance of implementing tailored methods to reach diverse dementia caregivers.

